# 2884. Incidence of Invasive Fungal Infections After Pancreas Transplantation

**DOI:** 10.1093/ofid/ofad500.161

**Published:** 2023-11-27

**Authors:** Vaisak Nair, Zachary A Yetmar, Maria T Seville, Wendelyn Bosch, Elena Beam

**Affiliations:** Mayo Clinic, Rochester, MN; Mayo Clinic, Rochester, MN; Mayo Clinic Arizona, Phoenix, Arizona; Mayo Clinic, Rochester, MN; Mayo Clinic, Rochester, MN

## Abstract

**Background:**

There is scarce data surrounding the epidemiology of invasive fungal infections (IFI) after pancreas transplantation (PT). Our centers’ PT protocols recommend universal fluconazole prophylaxis for at least 1 month, which is extended to 1 year for recipients who live in *Coccidioides* endemic areas. The purpose of our study was to evaluate the epidemiology of IFIs after PT while on our antifungal prophylaxis protocol.

**Methods:**

A retrospective cohort study was performed among adult patients who underwent PT at Mayo Clinic sites in Arizona, Florida, or Minnesota between January 1, 2010 and December 31, 2020. IFI was defined based on EORTC/MSGERC criteria. The primary aim was to describe epidemiology and timing of IFI after PT, particularly in the context of the universal antifungal prophylaxis protocol.

**Results:**

Among 482 pancreas transplant recipients, 35 developed IFIs (7.3%). All patients received antifungal prophylaxis per protocol for a median duration of 33 days (Figure 1). Median duration from time of transplant to IFI was 16 (IQR 10.5-28.2) days for *Candida* IFIs versus 529 (IQR 192.5-1195.0) days for non-*Candida* IFI. 20 IFIs (57.1%) were secondary to *Candida* species with 45% of these cases caused by either *C. glabrata* or *C. krusei* (Figure 2). 90% were on antifungal prophylaxis (85% fluconazole) at time of diagnosis of invasive candidiasis. All the *Candida* IFIs were intra-abdominal infections. Amongst the 15 non-*Candida* IFIs (42.9%), 9 were secondary to endemic mycoses and 4 were secondary to invasive molds.

**Figure:**
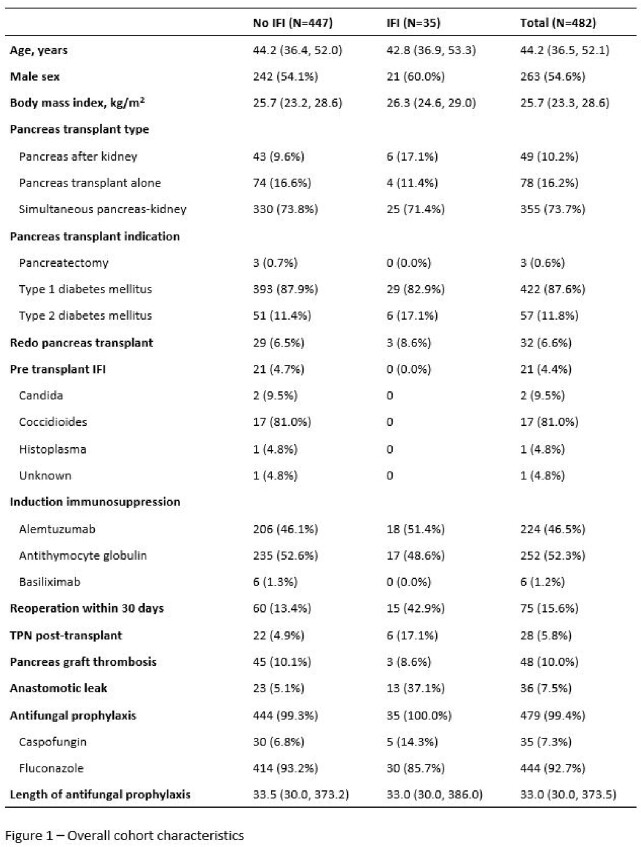

**Figure:**
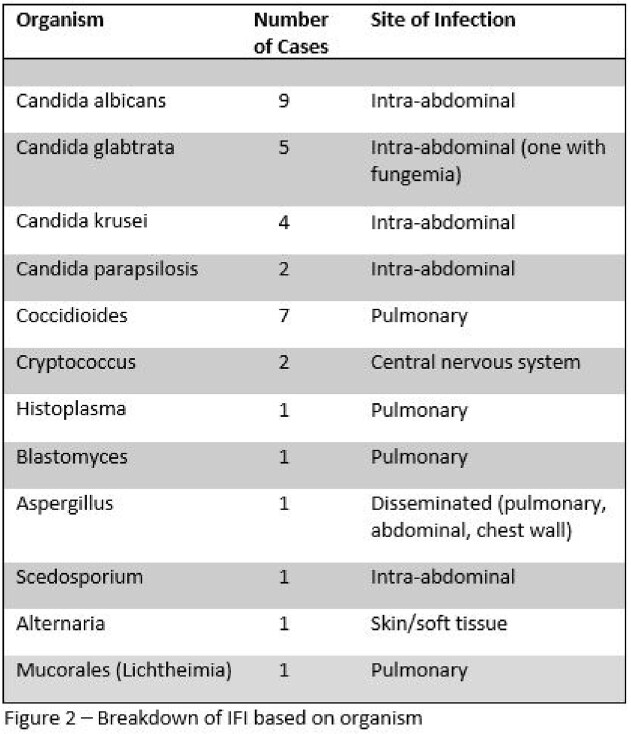

**Conclusion:**

Our study shows a high incidence of IFIs secondary to *Candida*, with few patients diagnosed with non-*Candida* IFIs (Figure 3). Non-*Candida* IFIs, mostly secondary to endemic mycoses, occurred more than a year after transplant. Patients are at highest risk for *Candida* IFIs within the first 30 days after PT, likely secondary to organ space surgical site infections, despite receipt of antifungal prophylaxis. Breakthrough invasive candidiasis occurred in 90% of cases. Though our study lacked a comparator group without prophylaxis, these data question the effectiveness of fluconazole prophylaxis after PT.

**Figure:**
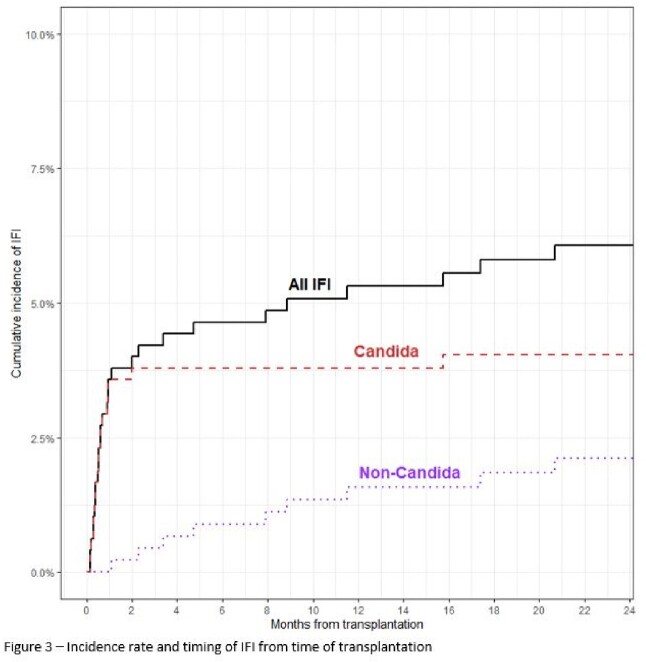

**Disclosures:**

**All Authors**: No reported disclosures

